# Assessing the Efficacy and Reliability of Pedicled Myocutaneous Single-Staged Flaps in Oral Cancer Ablation Surgery: A Prospective Interventional Study

**DOI:** 10.7759/cureus.77484

**Published:** 2025-01-15

**Authors:** Sapna D P Somani, Vivek N, Abinaya Subramaniam, Saravanan Chandran

**Affiliations:** 1 Oral and Maxillofacial Surgery, SRM Kattankulathur Dental College and Hospital, SRM Institute of Science and Technology, Chennai, IND

**Keywords:** locoregional, oral cancer, pedicled myocutaneous flaps, reconstruction, reliability

## Abstract

Background

Reconstructive ladder following cancer ablation surgery varies from the simplest of split skin grafts to the most complex vascularized free tissue transfer flaps.

Objectives

The study objective is to assess the efficacy and reliability of single-staged pedicled myocutaneous flaps (PMF) for small- and medium-sized cancer defect reconstruction, as well as to evaluate the indications of single-stage PMF in the current free flap era.

Materials and methods

This was a prospective interventional study that was conducted over a period of nine months. All patients who had undergone cancer ablative surgery resulting in a small- or medium-sized defect during the study period are included. Reconstruction was performed by a single-stage PMF. Postoperatively, periodic recipient and donor site assessments were done at an appropriate time interval.

Results

During the study period, 21 patients were reported for cancer ablation surgery. Of these, 19 patients underwent PMF reconstruction. The peak incidence of tumors was noted in patients having an age range of 51-64 years. The chi-square test was used to compare the categorical variables. Friedman test was used to compare the visual analog scale (VAS) mean score between the time intervals. The statistical significance was kept at a p-value less than or equal to 0.05. Out of 19 patients who were operated on, no patient had dehiscence or fistula formation. No patients reported severe pain or infection in the donor site. There was no statistically significant association between necrosis at any post-operative interval. The mean patient satisfaction score measured using the VAS was 8.2.

Conclusion

In the current era, microvascular free tissue transfer remains the top reconstruction option. Still, our study retracted that a single-stage PMF remains a viable reconstructive option owing to its easy harvest, versatility, reliability, fewer postoperative complications, short operating time, and mainly cost factor in developing countries due to patients' financial constraints.

## Introduction

Surgery, radiotherapy, and chemotherapy have long been considered the primary modalities of cancer treatment. Single-modality treatment is only possible when the malignancy is diagnosed at its early stage [1]. However, with advanced stages of disease, multimodality treatment is the gold standard [2].

A single modality treatment might suffice when the lesion is early and small, either through surgery or radiotherapy. However, with larger lesions, multimodal therapy may be warranted [1]. Though surgery remains the mainstay in head and neck cancers, radiotherapy, with its organ preservation advantage, might be considered the modality of choice in carcinomas involving lip, tongue, and buccal mucosa. Nevertheless, if the lesion becomes extensive or abutting the bone, surgery is the primary choice of treatment, followed by either postoperative radiotherapy or chemotherapy [2].

Surgical resection of any tumor leaves a large defect, especially in the head and neck area, which could significantly compromise the form and function [3]. Hence, following ablative cancer surgery, disfigurement of the face can pose a huge reconstructive challenge to both the surgeon as well as the patient.

Primary closure or healing by secondary intention might still be possible in cases of small defects. However, in the majority of cases, the defect warrants tension-free wound margins to achieve adequate wound healing without breakdown. The best reconstruction strategy for any head and neck defects should include an excellent cover and lining. The selected flap should mimic the resected tissues' type, thickness, texture, mobility, sensation, and function as closely as possible. Reconstructive ladder varies from the simple split skin grafts to the most complex microvascular free flap transfer [4]. 

The thoughtful analysis for choosing reconstruction options includes assessing the patient's health, economic status, average operative time, availability of resources, and skilled personnel [5]. Microvascular free tissue transfer is often chosen as the first choice in head and neck reconstruction. Free flap transfer involves the dissection and excision of a portion of skin, subcutaneous tissue, with or without the underlying muscle with its vascular supply. It requires anastomosis of blood vessels at the reconstructive site.

Reconstructive options were individualized for every patient based on the expected defect size. Large defect size requires free flaps, whereas small- to medium-sized maxillofacial defects can be reconstructed using local rotational, transpositional, or advancement flaps leading to excellent healing [6]. Defects were classified as small (up to 4 cm maximum diameter), medium (up to 7 cm), and large (more than 7 cm) [7].

Pedicled myocutaneous flaps (PMF) comprise a full or partial thickness of muscle, overlying skin, and subcutaneous fat attached by a vascular pedicle containing its blood supply. They are generally tunneled subcutaneously to a surgical site and rotated on the vascular stalk to the site of reconstruction. Commonly used myocutaneous flaps are the rectus abdominis flap as well as latissimus dorsi, pectoralis major, temporalis, sternomastoid, gracilis, gluteus, and sartorius muscle flaps [7]. 

The purpose of our study is to evaluate the indications and reliability of using PMF in the current free flap era and also to share a single-centric care experience. Though there are various reconstructive options, certain locoregional flaps might need a staged procedure for pedicle division. Hence, this study also evaluates the clinical versatility of single-stage PMF in functional and aesthetic rehabilitation of small- to medium-sized defects following cancer ablation surgery.

## Materials and methods

Study design

A prospective clinical observational study was carried out for nine months, from 1st December 2022 to 31st August 2023, including a minimum of three months of follow-up. SRM Institutional Ethical Committee approved this study (1792/IEC/2019), and informed consent was obtained from all patients. Participants who fulfilled European cancer guidelines and were willing to be part of the study were included. Each participant went through a systematic clinical, radiological, and histopathological assessment. The study was conducted. Patients requiring primary reconstruction following cancer ablation surgery were included in our study. Moreover, patients having large composite defects or those who had previously undergone irradiation therapy in the head and neck region were excluded. The final treatment plan was arrived at after discussion with the Institutional Tumor Board. Interdisciplinary opinions were obtained and recorded to discuss multimodality treatment options.

**Figure 1:**
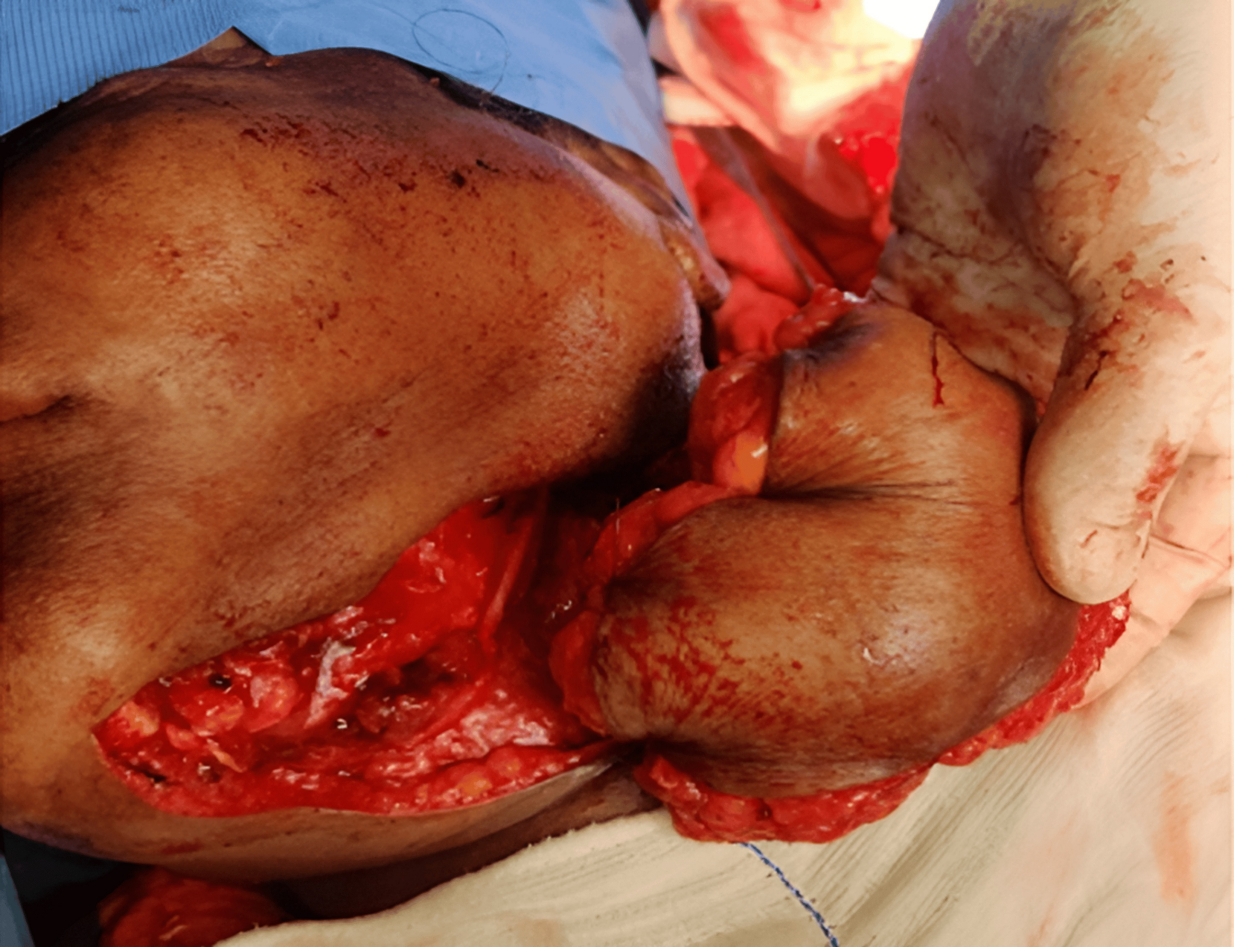
Pectoralis major myocutaneous (PMMC) flap reconstruction Image Credits: Dr Sapna D P Somani

**Figure 2:**
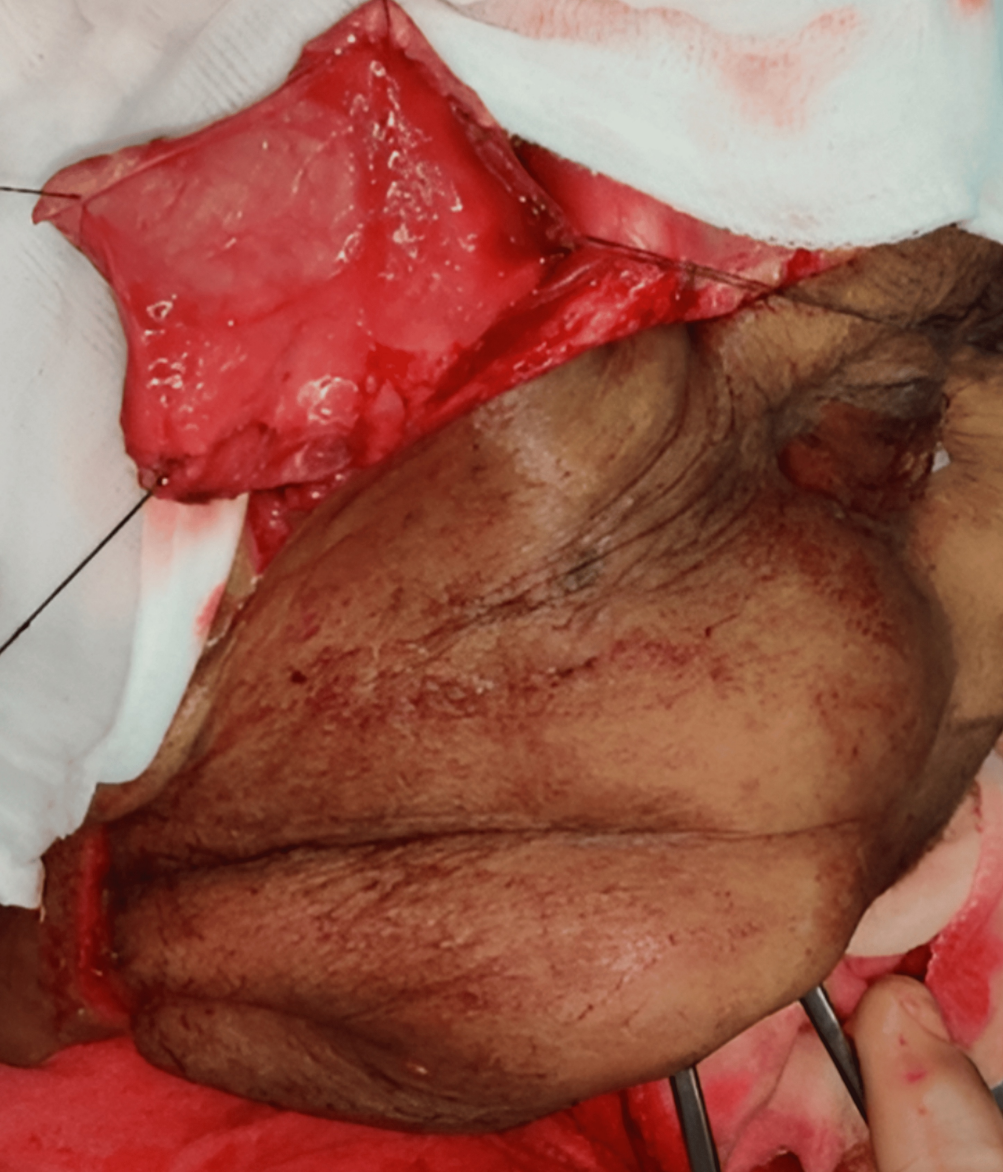
Temporalis flap reconstruction Image Credits: Dr Sapna D P Somani

**Figure 3:**
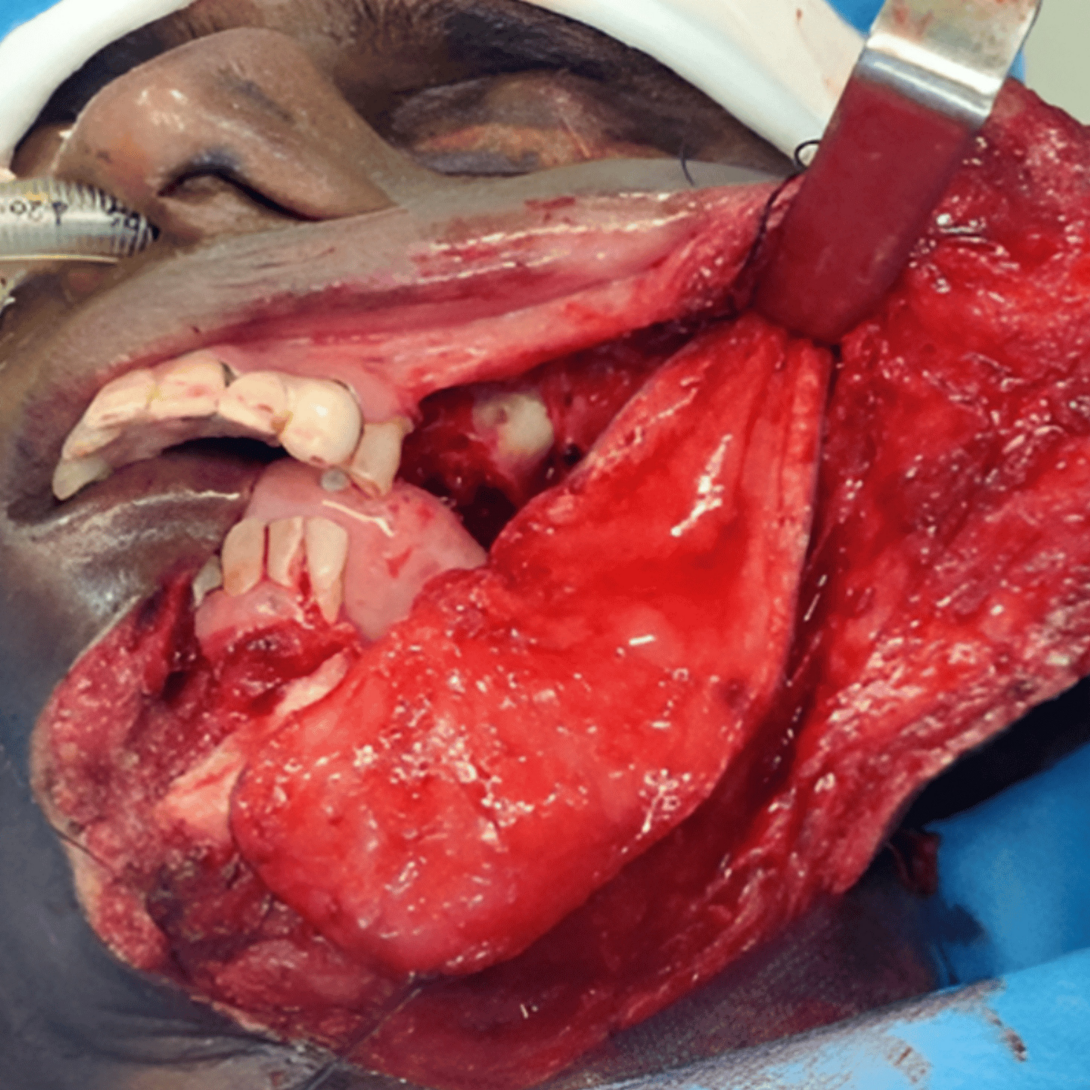
Forehead flap reconstruction Image Credits: Dr Sapna D P Somani

**Figure 4:**
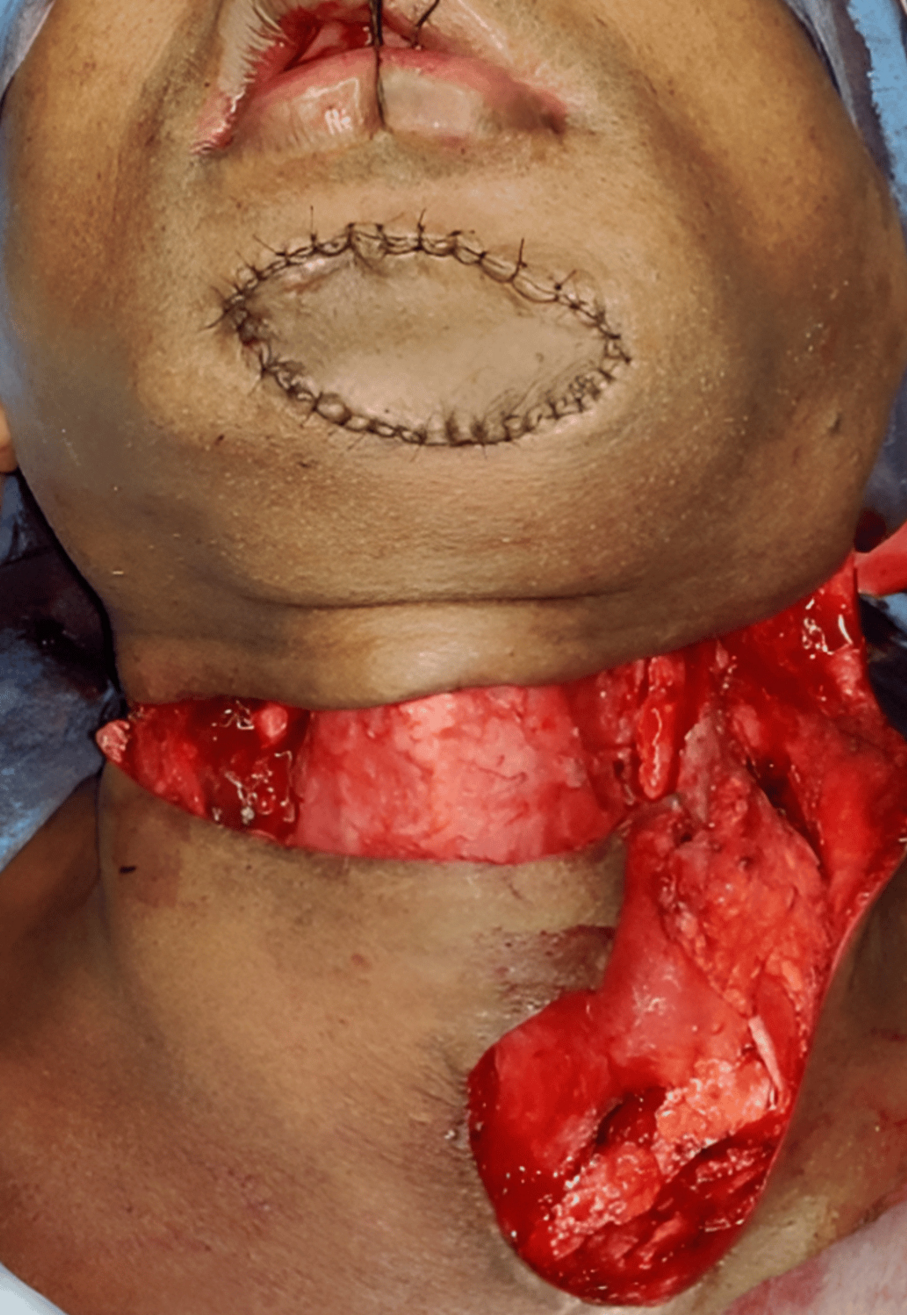
Sternicleidomastoid flap reconstruction Image Credits: Dr Sapna D P Somani

Reconstructive options were individualized for every patient based on the expected defect size. Defects were classified as small (up to 4 cm maximum diameter), medium (up to 7 cm), and large (more than 7 cm) [7]. Patients who came under small and medium defects were included for PMF reconstruction. Though all our patients had surgery as a primary modality of treatment, post-operative radiotherapy/chemo-radiotherapy (RT/CRT) was administered to patients depending on the final histopathological report. All patients had a periodic review schedule. Reconstruction was performed for 19 patients. A pectoralis major myocutaneous (PMMC) flap (Figure 1) was used for 16 patients, a temporalis and masseter muscle flap for one patient (Figure 2), a forehead flap for one patient (Figure 3), and an Abbe-Estlander flap along with a sternocleidomastoid (SCM) flap (Figure 4) for one patient.

Study procedure

Pre-operative Assessment

All patients underwent thorough clinical examination, including systemic evaluation. Patients reporting any suspected lesion would have a detailed clinical history and systematic clinical evaluation. Incisional biopsy under local anesthesia would be obtained and sent for histopathological assessment. Once histopathologically confirmed as malignancy, the patient goes through contrast-enhanced computed tomography from the base of the skull to the clavicle with chest screening to assess the extent of the lesion. The patient also undergoes all basic hematological assessment tests. Ultrasonography of the abdomen was done as a screening. After detailed assessment and counseling, the patient's records will be discussed with the interdisciplinary tumor board to arrive at the appropriate treatment plan.

Intra-operative Procedure

All surgeries were performed following the asepsis protocol. Following standard patient preparation protocol, as per the radiological and clinical evidence of disease and nodal involvement, either selective or modified radical neck dissections were carried out. Depending on the TNM tumor staging and the infiltration of the lesion into the surrounding structure, wide local excision with an oncological safety clearance margin was performed. An intraoperative frozen section was done to obtain a tumor-free margin clearance. After the margins were deemed cleared, the reconstructive team performed the planned loco-regional pedicled myocutaneous flap.

Postoperative Care

All patients were provided with intensive care for a minimum of three to five days. Vitals monitoring was done, the operation reconstructed as well and donor site care was provided. Adequate postoperative antibiotic, steroid, and analgesic coverage were given. Adequate mobilization, deep vein thrombosis (DVT) prophylaxis, chest and limb physiotherapy, and a high-protein liquid diet were provided. Postoperatively, periodic recipient and donor site assessment was done at an interval of post-operative (POD) day 1, day 7, day 15, day 30, and after three months. Once the final post-operative histopathology reports were obtained, patients were referred to a radio oncologist for further adjuvant chemo-radiotherapy.

Statistical Analysis

The statistical analysis was done using SPSS version 25.0 (IBM Corp., Armonk, NY, USA). The Chi-square test was used to evaluate between the categorical variables. The Friedman test was used to assess the visual analog scale (VAS) mean score between the time intervals. The statistical significance was kept at a p-value ≤ 0.05. The outcomes of this study are showcased based on the following parameters of recipient and donor sites.

## Results

In the course of the study, 21 patients reported to our outpatient unit with lesions of varying sizes and histologically proven squamous cell carcinoma. Out of 21 patients, two patients had microvascular free flap resection following cancer resection surgery (one with osteocutaneous radial forearm flap and the other patient with osteocutaneous free fibula flap) since the defect size was large (more than 7 cm). Nineteen patients with small (up to 4 cm maximum diameter) to medium-sized (up to 7 cm) defects were reconstructed using loco regional single-stage pedicled myo-cutaneous flaps. 

In our study, 70% of the total population were males, and 30% were females. Sixty percent of patients ranged within the 51-64 age group. Sixteen patients had a lesion in the buccal mucosa, two had intra-alveolar carcinoma (IAC) of the mandible (gingival, buccal sulcus (GBS)), and one patient had carcinoma of the maxillary antrum. 

Surgical outcome

No intraoperative deaths ocurred in this series. One patient died in the postoperative phase since he had tuberculosis. No major complication occurred in any patient.

Flap assessment

Vascularity and Necrosis

PMMC flap was used for 16 patients, forehead flap for one patient, temporalis and masseter flap for one patient, Abbe-Estlander flap, and SCM flap for one patient. All flaps were healthy and maintained vascularity without any change in color of the flap except the SCM flap. SCM flap had a change in color by POD 7, i.e. purple. The color had changed to black by POD 15. Complete necrosis of the SCM flap was seen by POD 15. The failed SCM flap was managed successfully with the PMMC flap. There was no statistically significant association between necrosis at any post-operative interval.

Dehiscence, Fistula, and Nerve Function

In the immediate postoperative period, four patients developed dehiscence, which healed by secondary intention. Patients who had reconstruction using the forehead flap had a transient loss of the frontal branch of facial nerve function with complete recovery within three months postoperatively (Table 1). Overall statistical significance was seen amongst the parameters of dehiscence in the postoperative period, which implies that the above said complication seen in the POD 7 to POD 30, subsided over a period of three months in all patients. It also implies the importance of appropriate postoperative clinical evaluation.

**Table 1:** Frequency and percentage distribution of presence or absence of dehiscence at follow-up time intervals * significant P-value

Variables			Dehiscence			Total	Chi-square value	P- value
			No	Yes	Not applicable			
Time period	Day 1	N (%)	21 (100.0)	0 (0.0)	0 (0.0)	21 (100.0)	10.571	0.032*
	After 7 days	N (%)	17 (80.95)	4 (19.04)	0 (0.0)	21 (100.0)		
	After 15 days	N (%)	19 (90.48)	2 (9.52)	0 (0.0)	21 (100.0)		
	After 30 days	N (%)	20 (95.24)	1 (4.76)	0 (0.0)	21 (100.0)		
	After 3 months	N (%)	20 (95.24)	0 (0.0)	1 (4.76)	21 (100.0)		

Speech and Oral Sphincter

Postoperatively, all patients were able to speak normally and clearly. The intelligibility was measured and recorded. Out of 19 patients, three patients had occasional drooling (Tables 2, 3). This occasional drooling had subsided in postoperative phase of three months. This parameter was statistically significant in the postoperative evaluation. The speech evaluation in postoperative phase improved after 15 days. Overall statistical significance in oral sphincter and speech implies that it improved during the postoperative phase. 

**Table 2:** Frequency and percentage distribution of oral sphincter at follow-up time intervals **significant P-value

Variables			Oral sphincter				Total	Chi-square value	P-value
			Normal	Occasional drooling	Continuous drooling	Not applicable			
Time period	Day 1	N (%)	2 (9.52)	18 (85.72)	1 (4.76)	0 (0.0)	21 (100.0)	20.769	< 0.001**
	After 7 days	N (%)	2 (9.52)	18 (85.72)	1 (4.76)	0 (0.0)	21 (100.0)		
	After 15 days	N (%)	19 (90.48)	2 (9.52)	0 (0.0)	0 (0.0)	21 (100.0)		
	After 30 days	N (%)	19 (90.48)	2 (9.52)	0 (0.0)	0 (0.0)	21 (100.0)		
	After 3 months	N (%)	19 (90.48)	1 (4.76)	0 (0.0)	1 (4.76)	21 (100.0)		

**Table 3:** Frequency and percentage distribution of speech at follow-up periods ** significant P-value

Variables			Speech					Total	Chi-square value	P-value
			Normal	Easily intelligible	Poorly intelligible	Unintelligible	NA			
Time period	Day 1	N (%)	0 (0.0)	0 (0.0)	2 (9.52)	19 (90.48)	0 (0.0)	21 (100.0)	30.667	< 0.001**
	After 7 days	N (%)	0 (0.0)	1 (4.76)	18 (85.72)	2 (9.52)	0 (0.0)	21 (100.0)		
	After 15 days	N (%)	10 (47.61)	7 (33.33)	4 (19.04)	0 (0.0)	0 (0.0)	21 (100.0)		
	After 30 days	N (%)	18 (85.72)	3 (14.28)	0 (0.0)	0 (0.0)	0 (0.0)	21 (100.0)		
	After 3 months	N (%)	19 (90.48)	1 (4.76)	0 (0.0)	0 (0.0)	1 (4.76)	21 (100.0)		

Diet Tolerance

All patients had received the diet via the parental route (nasogastric tube) during the first postoperative week. Following this, the oral diet was resumed gradually in all patients. All patients very well tolerated liquid and solid diet.

Donor Site Morbidity

The donor site was assessed for edge approximation, dehiscence, infection, and functional restriction. Partial dehiscence of the donor site was observed in one case, which had secondarily granulated within one month postoperatively. Out of 19 patients, primary closure of the donor site was done for 16 defects, while three patients required split skin grafting for the closure of the defect. No patient reported severe pain or infection in the donor site. 

Functional Restrictions

Depending on the site of the flaps harvested for defect closure, functional restrictions of respective donor sites were monitored at periodic intervals. They were categorized into none, mild, moderate, and severe. Out of 19 patients, seven patients had severe, and 12 patients had moderate restrictions in function on POD 0. However, after three months of follow-up, 17 patients had no functional restrictions, while two had mild restrictions in movement (Table 4). The statistical significance implies that all patients had improvement in functional restriction in the postoperative phase.

**Table 4:** Frequency and percentage distribution of functional restrictions at follow-up time intervals * significant P-value

Variables			Functional restrictions					Total	Chi-square value	P-value
			None	Mild	Moderate	Severe	Not applicable			
Time period	Day 1	N (%)	0 (0.0)	2 (9.52)	12 (57.14)	7 (76. 19)	0 (0.0)	21 (100.0)	63.295	0.000*
	After 7 days	N (%)	0 (0.0)	4 (19.05)	17 (80.95)	0 (0.0)	0 (0.0)	21 (100.0)		
	After 15 days	N (%)	0 (0.0)	17 (80.95)	4 (19.05)	0 (0.0)	0 (0.0)	21 (100.0)		
	After 30 days	N (%)	14 (66.66)	5 (23.80)	2 (9.52)	0 (0.0)	0 (0.0)	21 (100.0)		
	After 3 months	N (%)	17 (80.95)	2 (9.52)	1 (4.76)	0 (0.0)	1 (4.76)	21 (100.0)		

Patient Satisfaction

In our study, the VAS was used for the aesthetic evaluation of the flap. During the first week of the postoperative period, the mean score was 8.20, which eventually improved to 8.50 after one month postoperatively. The aesthetic mean score had improved to 8.90 three months postoperatively. There was statistically significant progress in aesthetics over three months. The statistical significance implies that all patients were satisfied with the aesthetic outcome of the flap (Table 5).

**Table 5:** Mean Visual Analog Scale score of recipient site esthetic analysis * significant P-value

Time period	Visual Analog Scale mean score (esthetic evaluation)	Chi-Square value	P-value
After 7 days	8.20	23.568	0.000*
After 15 days	8.50		
After 30 days	8.50		
After 3 months	8.90		

Statistical analysis was done for all recipient site parameters, which showed significance in patient satisfaction, speech, oral sphincter action, and diet tolerance (Table 6). This implies that there is no functional and esthetic compromise in the recipient site.

**Table 6:** Recipient site statistical analysis ** significant P-value

Parameters	Chi-Square value	P-value
Vascularity	4.000	0.406
Necrosis	4.000	0.406
Dehiscence	10.571	0.032*
Infection	4.000	0.406
Visual Analog Scale	23.568	0.000**
Speech	30.667	<0.001**
Oral Sphincter	20.769	<0.001**
Diet	38.269	<0.001**

## Discussion

Squamous cell carcinoma is the most frequently occurring malignancy, accounting for 90% of all oral and oropharyngeal cancers [2]. Though the etiology of any malignancy is probably multifactorial, the most accepted risk factors considered are tobacco and alcohol consumption [2]. Major risk factors considered responsible for the malignant transformation of tissues are the 6S's, i.e., smoking, spirit, spices, syphilis, sunlight, and sharp teeth. The severity of the disease is decided based on the duration of irritation or the causative factor responsible for the progression of the disease from carcinoma in situ up to poorly differentiated squamous cell carcinomas. In India, 60 to 80% of patients often present with advanced disease, as compared to 40% in Western countries [8].

The goal of surgery is the resection of the tumor with tumor-free clearance margins. Depending on the site of the tumor, the spread of the disease might lead to the involvement of adjacent soft and hard tissues. Resection of such an extensively spread disease necessitates wide local excision and composite resection involving mucosa, musculature, maxilla or mandibular cortices, and/or skin [5].

Over the years, there has been a great shift in the choice of reconstruction for small- to medium-sized defects. For more than two to three decades, free flaps have become the gold standard for the reconstruction of medium- to large-sized composite defects. The aim for reconstruction has shifted from mere defect coverage to aesthetic and functional rehabilitation [9].

Free vascularized flaps are popular due to their ability to replace each component of defect, i.e., muscle, bone, and skin, with available fasciocutaneous, fasciomyocutaneous, or osteomyocutaneous flaps. This provides excellent functional and aesthetic rehabilitation [9]. Nonetheless, these highly skilled free flaps require meticulous handling of tissues and optimal hemodynamics for excellent flap survival. Systemic comorbidities of patients are not always suitable for flap survival. Also, extensive hours under general anaesthesia, manpower, and the use of resources like microscopes make it difficult to reconstruct every composite defect [9].

According to the literature, locoregional flaps, on the other hand, manage to overcome the difficulties offered by vascularized free flaps and provide excellent defect coverage [6,10]. In 1979, Ariyan S used the pectoralis major myocutaneous flap to reconstruct four large defects and suggested that the PMMC flap is one of the most versatile locoregional flaps [10]. In 2011, Nayak and Nilamani reported that in head and neck regions, single-stage reconstruction with deltopectoral (DP) and pectoralis major myocutaneous flaps offers an alternative to microvascular procedure [11]. In 2015, Tripathi M et al. conducted a pilot study to compare two incision designs for PMMC flap harvesting: lateral axillary incision and without lateral axillary incision. They found that the latter technique yielded better results and was effective in terms of time of harvesting, time of closure of donor site, flap survival with minimal donor site complications, and improved outcomes [12]. 

Abubaker et al., 2002, reported that the temporalis flap could yield abundant tissue with minimal to no functional morbidity or aesthetic deformity in the donor site [13]. In 2003 Tanaka et al. showed that indications of sternocleidomastoid myocutaneous flap can be extended with increase in the number of supraomohyoid and functional neck dissection cases [14]. This proves that an adequate knowledge of locoregional flaps enables an ablative surgeon a better one in offering optimum patient care in a setup where a microvascular facility is not available [15].

Small- to medium-sized maxillofacial defects can be reconstructed using local rotational, transpositional, or advancement flaps leading to excellent healing [6]. Chen, in 2019, suggested that the Abbe-Estlander flap is ideal for reconstructing clinical stage II disease defects [16]. Sahu et al. documented an alternative technique in which the maxillectomy defect was covered with a pedicled forehead flap based on the parietal and frontal branches of a superficial temporal artery [17]. Jincai Fan, 2000, presented a new technique of Scarless Expanded Forehead Flap in conjunction with tissue expansion without visible scarring [18]. He suggested that the flap could be harvested as large as 8x18 cm without inducing flap necrosis or problems with donor-site closure.

Our study aimed to evaluate the versatility and reliability of single-stage pedicled myocutaneous flaps and to assess functional and aesthetic rehabilitation of small- to medium-sized defects following cancer ablation surgery. In the study period of nine months, 19 patients had reported to our department and were included in the study. 

In this study, out of 19 patients who had undergone resection and reconstruction, six patients suffered from various systemic illnesses like diabetes (two), hypertension (one), ischemic heart disease (one), and pulmonary tuberculosis (two).

One defect, which was reconstructed with an SCM flap, had reconstruction plate exposure, gradual loss of vascularity, and complete necrosis of the SCM flap. This patient had a history of pulmonary tuberculosis for which he had completed the course of anti-tubercular medications. After a month, the necrosed flap was excised, and the resulting defect was reconstructed using PMMC and DP flap. This flap was chosen due to its reliability and versatility in cases of limited resources and heavy patient load [16-18]. This makes PMMC a workhorse for the reconstruction of maxillofacial defects.

Sixteen patients had a lesion in the buccal mucosa, two had IAC of the mandible (gingivo buccal sulcus - GBS) while one patient was diagnosed with moderately differentiated carcinoma of left maxillary antrum. The tumor had involved most of the area around the orbit. Wide local resection of the lesion was done. The lateral orbital wall and floor of the orbit were removed and compartmental resection was done. Orbital contents were supported using temporalis muscle-coronoid swing flap reconstruction. The remaining defect was temporarily filled with impression compound. Later, a provisional obturator was given, and healing was assessed periodically. The patient had post-operative diplopia, which was reduced over a while and was later managed using bifocal lenses. In one patient, the temporalis flap, along with the masseter muscle flap, was used in carcinoma of buccal mucosa for reconstruction following resection. Post-operative results were satisfactory, with an inconspicuous scar and adequate mouth opening one month postoperatively.

In our study, all patients received post-operative CT and RT or CRT. The aesthetic outcome was statistically significant. Most of the patients were able to return to their profession and daily life with social acceptability. 

Common complications observed in the donor site were partial dehiscence which had secondarily granulated within one month post-operatively. One patient who had reconstruction using the forehead flap had a transient loss of the frontal branch of facial nerve function with complete recovery within three months post-operatively.

Limitations of the study

This study mainly projects the single center experience of challenging cases reconstructed with single staged pedicled myocutaneous flaps operated over a period of nine months, which justifies the smaller sample size of the study. Our study also had less heterogeneity in the sample, with 70% of cases being carcinoma of the buccal mucosa. All the flaps except one survived postoperatively, as well as during the further follow-up period. Another limitation of the study is short follow-up for three months. Better assessment of flap condition requires long-term follow-up for at least a year. 

## Conclusions

To conclude, the single-staged pedicled myocutaneous flap remains an essential reconstructive option in the head and neck region because of its technical ease, reliability, clinical versatility, function, aesthetics, short operating time, and reduced postoperative complications in donor and recipient sites. In the current era of free tissue transfer, due to limited medical resources and patients' financial constraints in developing countries, single-stage PMF will remain irreplaceable.
